# The Teeth of Children

**Published:** 1894-02

**Authors:** Howard Van Antwerp

**Affiliations:** Mt. Sterling, Ky.


### The Teeth of Children.

BY HOWARD VAN ANTWERP, D.D.S., MT. STERLING, KY.

*Gentlemen of the Kentucky Association:*

The opinion that our professional practice should be more largely preventive than it is and the proverbial ratio between prevention and cure, suggested to me the consideration of the above subject, along the line of their structural improvement, by the employment of a more rational dietary. Observation has further convinced me of the importance of an investigation of this phase of the subject. That there is need of some radical change looking to the better development and more perfect calcification of the teeth of these young patients is evident to the most casual observer in the profession. We are almost daily appealed to in this wise: "Doctor, why are Jennie's teeth so bad, when her father's and mine are good yet?" or "my teeth were not decayed so early, why is it?" As a rule the dentist questioned, through ignorance or a desire to avoid an explanation of "the why," ascribes it, in a general way, to "modern civilization," "race degeneracy," or something else equally indefinite. True enough, but of what practical help? Nothing is done to check the evil or to prevent it in succeeding progeny. Parents are not so much to blame, because of lack of education along this line, and for the dentist appealed to, to fail to answer these anxious questions in a definite and helpful manner is, at least, reprehensible practice.

The consideration of the subject will be from a common-sense standpoint and I trust physiological. Not having enjoyed (?) the supervision of a nursery, I cannot give you the result of personal experience in the natural manufacture of good teeth, but I believe they can be made good and hard. Modern dietetics fail to meet the requirements of the case and I would respectfully urge the point that the dentist is the proper person to intelligently direct and promote this change.

In the Dental Museum of the University of Michigan is to be found a fine collection of human maxillæ, skillfully prepared and arranged in series, showing their development and the calcification and eruption of the teeth. An examination of these preparations shows that at seven and one-half months, in utero, the jaws are almost as thin as paper with the crowns of the superior and inferior temporary centrals partially formed, with the other temporary teeth indicated by small pin-head nodules. At birth the dentine crowns of these centrals is well formed, while the crowns of the temporary molars are barely outlined. Eight months after birth we see the crowns of both upper and lower incisors fully formed, with their enamel complete, beginning to erupt and about one-third of the root portion formed.

At this time the first molar is just beginning to calcify. At one year the upper incisors and lower centrals erupted, roots half formed. First molars existing as flat, hollow caps. First trace of upper first centrals. Twenty months see all but temporary second molars erupted; temporary cuspids half through, while roots of temporary incisors are complete, and crowns of second temporary molars formed. Crown surfaces of first permanent molars well developed, fissures deep and cusps well defined. Crowns of upper and lower centrals and cuspids, permanent, well along.

At two years all of the temporary set are *fully* erupted except the second molars. First permanent molars in good position and coming, with half of crown full sized.

At four and a half years temporary set is complete, second incisors being in. Crowns of permanent centrals and cuspids fully formed but not completely calcified. Bicuspids beginning to form. First molar crowns fully formed and almost completely calcified.

At six years all temporary teeth present with roots fully formed. First permanent molars fully erupted with roots half formed. Central roots complete, grading back to bicuspid crowns, which are just shaping. Second molars are seen, crown shells full shape.

At *seven years* of age the *enamel* of all the *permanent teeth* (except the third molars) is *fully formed, even* the second molar *crown* being complete, though the roots are yet forming. The upper centrals are erupted as are also the lower incisors. The bicuspid roots are forming synchronous with those of the second molars, the roots of first molars being three-quarters formed.

Ten years of age finds these first molar roots complete, with the crowns of the second molars partially or fully erupted. Upper thirds just beginning to form.

At thirteen years all except lower second bicuspids and third molars are in.

At eighteen all the teeth except these thirds, are in place, in large, well-developed jaws. The greatest development of the maxillæ seems to occur between the second and fourth years, doubling their size during that period.

A resume of this table of calcification shows that so far as the *structural* formation of the permanent teeth is concerned, the die is cast by the time the child is ten years of age. During this time the entire osseous system has also been developing rapidly, but, unlike the other osseous structures, the teeth at this age are full size, and there can be no further growth in them to make up for any existing *structural* deficiency. How very important then it is that the dietary of children should contain an abundance of the elements needed by nature to build good sound teeth, free from those well-known peculiarities of form and texture that make them an early and easy prey to the ravages of caries. These surface indications are atrophied spots and girdles, soft, white belts and pits, with deep and irregular fissures, often decaying immediately after eruption, clearly exhibiting very faulty calcification of the enamel and the dentine as well.

A little girl has recently come under my care whose teeth well illustrate this theory, as well as the perversity of dental organization in the same family. Her father is quite old and never had anything done to his teeth—which he claims is the reason they are all there. Her mother's teeth are good to-day, some few filled, but they are very hard and of firm attachment. A brother, twenty-seven years old, has all thirty-two teeth, only three of which have been filled; they are remarkably well formed and very hard. Now about her own teeth, six upper anterior teeth filled three years ago. First molars filled "way back yonder," to use her own words. Six lower anterior teeth filled recently, as were the bicuspid fissures and sundry deep pits on the faces of the cusps of cuspids and bicuspids. She is just erupting her second molars—all decayed—the lowers especially presenting cavities that will sacrifice at least half their crown surfaces. Now the question is, whether or not this condition is due to a diet deficient in mineral matter. An inquiry into the dietary of this family shows: Father and mother raised up on old-fashioned water-ground wheat flour, Kentucky cornmeal and hominy, brown sugar and plenty of milk. The son has always been very fond of milk, and has eaten a great deal of corn bread. All are tall and possess large frames. The little girl—pet of the family—has rejected the coarser and plainer fare and subsisted almost entirely on hot biscuits and coffee, with sweetmeats to vary the monotony. The condition of her teeth is not to be wondered at, considering their opportunities.

Starting from the very beginning, we quote from Dr. W. X. Sudduth, in the "*American System*": "All cells lie embedded in or are bathed by a fluid which is more or less gelatinous in consistency. It is from this surrounding medium that the cells derive the supply of nourishment necessary for the performance of their functions. Cells are capable of cellular activity in proportion to the amount of cell-pabulum this fluid contains. I do not say that cells are *active* according to the amount of food supply present, but that they are *capable* of putting on cellular activity just in proportion to the amount of cell-food at hand, and in this way are stimulated to increased functional activity. Bricks cannot be made without straw, neither can tissues present increased functional powers without plenty of food with which to nourish themselves." And it is just so—nature cannot produce good teeth unless she has the necessary materials on hand during their period of production.

Van Bibra's table gives the composition of human teeth as follows:

| | CEMENTUM. | DENTINE. | ENAMEL. |
|---|---|---|---|
| Organic | 29.27 | 28.70 | 3.59 |
| Inorganic | 70.73 | 71.30 | 96.41 |

The inorganic portion divides up as follows:

| | DENTINE. | ENAMEL. |
|---|---|---|
| Phos. Cal | 66.72 | 89.82 |
| Carbon. Cal | 3.36 | 4.37 |
| Phos. Mag | 1.08 | 1.34 |
| Salts, etc | .83 | .88 |
| Organic | 28.01 | 3.59 |
| | 100.00 | 100.00 |

The totals of the first table give 238.44 parts of inorganic as against 61.56 organic—a proportion of four of inorganic to one of organic in these portions of the tooth. The composition of the enamel, the most important part of the tooth, because most exposed and least likely to repair, shows a very great preponderance of the inorganic: 96.41 inorganic to 3.59, organic; which last some writers even question as organized organic matter.

Now, how can this enamel, almost wholly mineral, be produced in the requisite perfection of form and structure, from food stuffs that contain but little if any of the necessary material?

In recognition of the disparity of opinion in regard to this theory, I wish to quote Bart: "The food supplies to the organism may be so managed as to secure very definite results. By increasing or diminishing the whole amounts of food ingested, by variations in the quality and character of them, and by the employment of some special and restricted methods of feeding, cures are effected not attainable by medicinal (or we might as truly say, '*operative*') treatment."

And, again, Dr. Shepard, of Boston, has said: "We must either deny the truth of the testimony of the most eminent chemical and physiological investigations or grant that the question of food lies at the foundation of all improvement of the tooth structure of the coming generations."

If there be a system of waste and repair that applies to the fluids of the body, to adipose and connective tissues, to membranous and fibrous portions, to cartilage, hair and nails, the natural inference is that waste and nutrition as a principle of life and growth applies as well to the harder structures—bone, dentine and enamel. To exclude a set of organs given such an important place and office in the human economy, from physiological processes common to the other tissues, is evidently unwise. Besides, no organs of the body so quickly show loss of nutrition as the teeth, clearly indicating a close dependence on nutritive processes for the maintenance of their integrity.

Now, with the chemical formation and the early period of development in mind, and the belief that *nature* does use the material at hand, let us consider what foods are best calculated to secure the proper development and perfect calcification of the teeth.

The suggestions made are as practical as possible under the circumstances. When asked what articles of diet are proper, we should have them at our tongue's end, manner of preparation, and all. A very limited observation will satisfy any one that the prevalent ignorance on the part of cooks in kitchens, and women who superintend cooking and order meals for large families of children is something awful to contemplate, and formidable to attempt to enlighten. However, we have our part in the play, and the satisfaction of duty done is most often our only reward.

On the supposition that the family physician is one of broad intelligence, we will confine ourselves to the food of the child after it has been taken from its exclusive milk diet. Milk secreted from healthy blood meets all the requirements, and less harm is done at this time than later. But during and after this time mothers too often think that their children cannot eat any solid food, and a slop diet is the result, affording little of the exercise necessary for the proper development of the maxillæ.

Milk is good, however, and should be given to growing children as often as desired—not allowing them to drink it fast or in very large quantities.

Of the solid foods, bread, "the staff of life," is our sheet anchor in this proposed reform; not the pure white loaf, demanded by the modern æsthetic palate, but the good, old-fashioned brown bread, tough and sweet. From the standpoint of chemical fitness, Graham, or whole-wheat flour, stands pre-eminent, as possessing more of the constituent elements of dentine and enamel than any other article of daily consumption. In the production of the popular high-grade flours of to-day, the most important part to the growing child is rejected; giving it muscle and fat, but no bone to put it on or teeth wherewith to grind. Graham flour possesses physical advantages. By its bulk and coarseness it distends the intestines and stimulates peristaltic action, producing a laxative effect. Besides promoting digestion in this way, being more granular, when eaten warm or even hot, it does not ball up in pasty, impermeable masses, its flaky nature giving the digestive fluids easy penetration. It makes a bread which has a sweet, wholesome taste, and which rarely fails to charm the appetite of the youngster.

For breakfast you can have cracked wheat or Graham flakes, with cream and a little sugar; or Graham cakes, browned to the "Queen's taste;" or Graham gems, moist and sweet; or Graham biscuits, rolled thin and baked brown and crisp.

For dinner you may have, occasionally, Graham muffins, so light that they seem to melt in your mouth. Then you have on hand all the time, the staple lunch, brown bread, yeast or "salt-rising," sweetened or plain loaf, good and tough, giving the teeth and jaws plenty of exercise. In these and a dozen other palatable forms, this great bone and tooth-making staple can be presented to the children, so that they will learn to love it. It is not wise to attempt to keep children on this food all the time, but as you desire them to possess good, sound teeth, give it to them as often as possible. In the preparation of the Graham breads the flour should always be sifted, rejecting the coarse bran as more injurious than helpful.

Then, too, we have the various forms of the whole-oat grain, almost as good a tooth-former as the wheat. Plain oatmeal, rolled or crushed oats and others, with many recipes for their palatable preparation. A mixture of rolled oats and "wheat germ" (so-called) two to one, makes a fine breakfast dish. And last, of the ordinary grains, we mention corn, if you please. That which makes Kentucky Spiritus Frumenti famous the world over; that which, with our beloved bluegrass—possessing this same subtle something—makes our horses; and that which chiefly sustains the "colored population" and gives them good teeth. While not classed very high in the list of bone-producers, the writer cannot help but believe that Kentucky corn is different from that of other States. An examination of many sets of teeth in the mouths of young negroes has shown them uniformly well placed, well developed, smooth and hard. It may be due to their coarse diet or to the chemistry of the corn, their chief article of diet. Certain it is that the country darky's teeth are as far ahead of his town cousin's who has "always staid with white folks," as said cousin's are ahead of his employer's more favored (?) children.

Beans are good, as are also potatoes, except in the spring, when they greatly deteriorate. Macaroni is also to be recommended, being placed very high on the list by some writers.

In closing this paper I should like to ask for a free and unsparing expression of opinion from the older members of the profession, in regard to the possibility of bettering the structure of these teeth. While we have considered them as children's teeth, yet they are to serve for scores of years, perhaps, and should be as good as possible to start with. To this end I have tried to direct your thoughts, and I trust shall have stimulated your efforts.

